# Analyzing the coevolution of interorganizational networks and organizational performance: Automakers’ production networks in Japan

**DOI:** 10.1007/s41109-017-0024-5

**Published:** 2017-02-28

**Authors:** Petr Matous, Yasuyuki Todo

**Affiliations:** 1 University of Sydney, Sydney, Australia; 2 University of Tokyo, Tokyo, Japan; 3 Waseda University, Tokyo, Japan; 4 Research Institute of Economy, Trade, and Industry, Tokyo, Japan

**Keywords:** Stochastic actor-oriented models, Interorganizational network evolution, Interorganizational network diffusion, Model validation, Japanese production networks

## Abstract

Organizations create networks with one another, and these networks may in turn shape the organizations involved. Until recently, such complex dynamic processes could not be rigorously empirically analyzed because of a lack of suitable modeling and validation methods. Using stochastic actor-oriented models and unique longitudinal survey data on the changing structure of interfirm production networks in the automotive industry in Japan, this paper illustrates how to quantitatively assess and validate (1) the dynamic micro-mechanism by which organizations form their networks and (2) the role of the dynamic network structures in organizational performance. The applied model helps to explain the endogenous processes behind the recent diversification of Japanese automobile production networks. Specifically, testing the effects of network topology and network diffusion on organizational performance, the novel modeling framework enables us to discern that the restructuring of interorganizational networks led to the increase of Japanese automakers’ production per employee, and not the reverse. Traditional models that do not allow for interaction between interorganizational structure and organizational agency misrepresent this mechanism.

## Introduction

It has been recognized that the system of industrial production cannot be realistically reduced to the behavior of individual organizations, quantifiable by traditional atomistic approaches. Network conceptualizations of interorganizational relationships have become widely accepted (Borgatti and Foster 2003; Lomi and Pattison 2006; Trapido 2013; van de Bunt and Groenewegen 2007). It has also been recognized that interorganizational networks are not stationary and that individual relationships do not exist in isolation. In other words, interorganizational networks evolve, and their structure influences their own dynamics (Ahuja et al. 2009; Gulati and Gargiulo 1999; Madhavan et al. 1998). However, methods used in empirical research on interorganizational networks still generally require the troubling assumption that outcomes or network structures are endogenous and that the researched systems are stationary or in equilibrium conditions (Ahuja et al. 2011; Lomi and Pallotti 2012; van de Bunt and Groenewegen 2007).

Since the first studies in the field, networks in organizational research have traditionally been treated either as independent variables explaining organizational outcomes or, less often, as dependent variables of organizational processes (Ahuja et al. 2009, Borgatti and Foster 2003). Until recently, the available tools were unable to simultaneously treat dynamic networks as both dependent and independent variables in organizational processes, and therefore, researchers had to adopt one of two perspectives: either assume environmental/structural determinism or emphasize organizational strategy and individual agency.

Using an example of dissolving Japanese keiretsu networks, the aim of this paper is to illustrate one way in which these two perspectives in organizational research can be united, thereby allowing dynamic analyses of organizations that both shape and are shaped by their networks. We utilize unique longitudinal survey data on supply relationships among Japanese automobile manufactures and apply novel model validation tests to demonstrate how to rigorously assess (1) the dynamic mechanism by which interorganizational production networks are formed and (2) the role of the dynamic network structures in organizational performance.

## Interorganizational structures and performance in Japanese automotive keiretsu networks

### The evolution of Japanese keiretsu networks

The choice of the empirical case for this article was motivated by reports of the changing structure of keiretsu networks in the Japanese automobile manufacturing sector and speculation concerning the consequences of this change for the performance of the sector (Shirouzu 2015). The automotive sector is a suitable target for learning about organizations in mature modern industries with vertically integrated production processes (Lomi and Pattison 2006). We focus on the supply network aspect of the Japanese automakers’ interorganizational networks because supply relationships are crucial for organizations, particularly automobile assemblers and manufacturers, that subcontract a large proportion of their production (Dyer 1996). Studying supply relationships allows researchers to uncover the complex interorganizational structure of national production systems (Sako 2004).

The keiretsu system is a special type of network organization of production that allows the participating corporations to outsource essential tasks to interlinked long-term partners that are “embedded” in strong relationships of mutual social obligations (Granovetter 1985). The keiretsu system has been widely adopted by Japanese automobile manufacturers and credited for the success of these corporations. In 1939, Toyota organized its first-tier suppliers into an official association and refused to deal directly with suppliers outside of this group. Toyota’s approach was supported by the Japanese government and soon imitated by other Japanese automakers (Wada 1992). Logistics and quality control were significantly simplified because parts were obtained exclusively from a small number of proven intermediating partners. Such arrangements allowed Japanese auto manufacturers to reduce lead times and manufacturing costs and achieve high volumes of external production with very lean purchasing departments (Kamath and Liker 1994; Wada 1992). During the high-growth period of the Japanese economy, resource procurement through a small number of mutually cooperating suppliers was praised for its effective information-sharing, for reducing the costs of monitoring, and for keeping associated revenues within a narrow circle of companies (Cooper and Yoshikawa 1994; Handfield and Bechtel 2002; Holmstrom and Roberts 1998; Lamming 2000). Furthermore, close links among keiretsu suppliers were understood to prevent their exploitation by overly powerful clients (Holmstrom and Roberts 1998).

After Japan fell into recession, some commentators predicted that keiretsu would become even stronger in the harsher economic environment because of the networks’ role in diffusing risk. Others opined that the keiretsu model had become outdated for a modern economy in which the challenge was no longer to secure access to resources and maximize production but had shifted to competing for limited demand by increasing efficiency and lowering prices (Ahmadjian and Lincoln 2001; Lincoln and Gerlach 2004). Qualitative accounts suggest that keiretsu have been yielding to market mechanisms and open competition in response to the shift in global demand to price-conscious consumers in emerging markets. Reportedly, some Japanese manufacturers have begun recommending that their suppliers develop new links with customers from other trading groups instead of relying solely on one main client (Lamming 2000).

Even the Toyota Group, the archetypal pioneer of the vertical keiretsu structure and currently the most profitable and best-selling automaker in the world (Kubota 2015; Toyota Motor Corporation 2014), is reportedly reconsidering the traditional arrangement. The former vice-president of the group publicly criticized its keiretsu suppliers for abusing their guaranteed exclusive position as brokers and for reselling parts produced cheaply by firms outside of the group at unjustifiably high margins (Shirouzu 2015). Reports have also emerged of interlinked suppliers colluding to drive their prices beyond reasonable levels to inflate their revenues (Shirouzu and Shiraki 2014). The need for lower procurement costs and less waste across the supply chain likely intensified during the global financial crisis, when safe haven demand inflated the value of the Japanese currency. The Japanese yen strengthened from 120 yen per US dollar in 2006 to 76 yen in 2011, making cars produced in Japan more expensive on global markets. This research is based on data from Japanese automakers during this turbulent period (2006–2011).

### Intermediation, closure, embeddedness, and performance

Most of the available research on the role of interorganizational keiretsu networks appears to focus solely on their structure without examining how the networks affect the economic performance of their members (Ingram and Simons 2002, Khanna and Yafeh 2005). Identifying affiliation with a keiretsu group is not straightforward (Yafeh 2002). Keiretsu groups are generally characterized by dense relationships among a set of companies, and the extent of a firm’s connections to other members of the group defines the firm’s affiliation. The distinguishing characteristic of keiretsu supply structures is that firms obtain components and materials via a limited number of long-term partners in their business group that function as intermediaries, rather than reaching out directly to numerous original producers.

It is unclear whether it is advantageous to interact with diverse suppliers through a smaller number of long-term intermediaries (Hoetker et al. 2007). From the perspective of disaster resilience, more extensive supply networks may experience more shocks but recover faster (Todo 2016). From the perspective of innovativeness, interacting only with long-term partners may lead to cognitive lock-in (Gargiulo and Benassi 2000). Moreover, having few trading partners decreases a firm’s autonomy. However, strong and trusting relationships between buyers and sellers may be preferable when low-modularity parts must be supplied, which may require close cooperation and risk-sharing in customizing each model (Hoetker et al. 2007). A lack of modularity may be one of the reasons that keiretsu networks were traditionally much stronger in the Japanese automotive industry than in the electronics industry (Lincoln and Gerlach 2004). In the electric machinery industry, standardized parts can frequently be bought off the shelf, and major Japanese electronics producers are reportedly more inclined than automakers to deal at arms’ length with a large base of suppliers (Asanuma 1989).

Yang and Babich (2015) note that intuition suggests that utilizing intermediaries for procurement should be the correct choice because of the superior knowledge that these intermediates possess regarding real supply costs. Intermediaries that match with multiple suppliers and multiple buyers improve supply chain performance, according to Belavina and Girotra (2012), by facilitating the adaptation of the supplier base to the buyers’ needs and by providing sufficient levels of business to the suppliers. However, these advantages do not apply when an intermediary (e.g., a first-tier supplier) serves only one client (the automobile assembler). Firms and entire industries in intermediary brokerage positions can exploit their partners through the market power that such positions provide (Talmud 1994). From the “structural holes” perspective (Burt 2000), it appears rational for firms that are connected only through an intermediary to “close” such holes by direct connections, but the dynamics of such network processes have rarely been investigated in the context of supply chains (Ryall and Sorenson 2007).

It is not well understood whether it is beneficial for an organization to focus its supply relationships on a small number of intermediating partners within its own business group or to diversify its relationships to a variety of organizations. The most robust (although not unchallenged) empirical evidence seems to favor the view that a major function of business groups in Japan in the 20^th^ century was risk sharing and revenue redistribution from high-performing firms to partners experiencing temporary difficulties (Khanna and Yafeh 2005). Using more recent data, He et al. (2013) support this view of the function of business groups in China, showing that the diffusion of shocks within groups decreases a given member firm’s risk of default.

In summary, two important streams of theoretical views can be found in the literature on interfirm networks. The first stream is based on conventional economic market theory and posits that markets consist of dynamic arm’s-length transactions between independent organizations. According to this view, it is efficient for firms to distribute their resource transactions over a large number of partners and frequently change these partners as needed (Baker 1990). This position is also consistent with resource-dependency theory, as it is considered rational not to depend on any single organization but rather maintain access to a large number of substitutable partners (Uzzi 1996).

As a reaction to these under-socialized economic views of interorganizational relationships, economic sociologists have proposed a social network view of interorganizational relationships. Social network literature focuses on relational considerations between organizations, which were neglected in the original economic views of the market or considered only in their dyadic form in transaction cost economics (Romo and Schwartz 1995).

Uzzi (1996) demonstrated that it may be beneficial for firms to develop strong relationships “embedded” in a dense network of transactions among a small number of long-term partners. The advantage of exclusive long-term relationships to organizations embedded in close-knit groups is trust, transfer of fine-grained information, and joint problem-solving arrangements. Cliques of embedded ties provide opportunities for resource pooling, cooperation, coordinated adaption, and the induction of network partners to share economic benefits (Uzzi 1996). White (1981) proposed that in competitive production markets, we should observe interfirm structures to gravitate toward dense cliques of producers watching each other.

Despite their labels, both “the network” and “the market” theoretical view of interorganizational transactions can be conceptualized as networks. In embedded organizational networks, links have longer duration, the number of partners is smaller and they tend to be densely interconnected with each other. In conventionally conceived ideal markets, links are considered highly dynamic, and actors may directly access any number of partners through arms’ length transactions. Regardless their dynamicity and structure, both arrangements and their consequences for performance can be rigorously empirically analyzed with network methods.

Although it may not be clear whether an arm’s-length market approach or a closed-network approach is more efficient, theories predict that in one market, firms will copy the strategies of their more successful partners and that the whole system will therefore converge toward the more efficient arrangement (Uzzi 1996).

## Unbundling interorganizational network effects

### Endogenous network evolution

A firm may be more likely to conduct business with another firm if the two already have common business partners (Matous and Todo 2014). Embedded ties emerge from such third-party referral networks, which transfer expectations of behavior from existing embedded relationships to newly matched firms (Uzzi 1996). Firms may also prefer suppliers that have no trading relationships with one another to prevent collusion. If such tendencies exist, interorganizational network structure effectively constrains its own evolution (Koskinen et al. 2015; Stuart 1998). The effects of interorganizational network structure on its own evolution is worth exploring in its own right, but it is also a source of endogeneity that must be controlled for in studying the relationship between the dynamics of networks and their outcomes (Ahuja et al. 2011). Furthermore, network dynamics may be influenced by suppliers’ and clients’ revenue dynamics. Assemblers with greater sales volumes may have to expand their supply chains, and more financially successful suppliers may be more attractive. Similarity in performance may also matter – successful companies may tend to cluster together – and researchers should also control for such a possibility.

Causality may operate in both directions. Changing performance may influence networks, which may in turn influence performance. If revenue and the number of suppliers are correlated, it may be that the supply network structure is the source of the increased performance rather than vice versa. Untangling the underlying mechanisms is challenging but important. If certain types and shapes of interorganizational networks are found to be associated with high organizational performance, it is important to understand whether it is a case of, for example, a certain type of supply network structure stimulating revenues (which would be a recommendable strategy for other ambitious firms to replicate), or whether only firms that are already successful tend to consequently reshape their supply chains in a certain manner (which would not necessarily provide practical lessons for other companies).

### Network consequences: topology versus flows

Organizational researchers explain network effects in terms of either topology or flows (Borgatti and Foster 2003). Within the former (“structuralist”) tradition, actors’ performance is examined in relation to the patterns of their ties to others (while neglecting the characteristics of the actors’ partners) (e.g., Burt 2000). Within the latter (“connectionist”) tradition, network links are conceptualized as conduits along which influence flows between the partners and the focal actor (e.g., Davis and Greve 1997). Whereas structuralists are concerned with the shape of networks around focal actors (e.g., is the number of suppliers related to performance?), connectionists are concerned with influence the of the partners’ characteristics on the focal actor’s characteristics (e.g., does suppliers’ performance affect clients’ performance?). In the present study, we address both perspectives on network consequences, while controlling for endogeneity.

### Hypotheses

Based on the reviewed network theories of interorganizational transactions, we formulate the following hypotheses regarding the structural embeddedness of interfirm relations (Uzzi 1996).

The first hypothesis concerns the dynamics of interfirm network evolution (H1): “Firms will prefer to maintain relations with established partners within their cliques.”

The second hypothesis concerns the relations between interfirm network topology and organizational performance (H2): “Firms that focus their transactions in a small number of important relationships will outperform firms that spread their transactions among a larger number of partners.”

The third hypothesis regards the flows of support between firms and organizational performance (H3): The relationships between firms are not purely transactional but facilitate learning and include transfer of important information and support in times of need. Therefore, connections with successful firms will increase the probability of good performance and decrease the probability of bad performance in the long term.

## Supply network data and firm performance measures

The analysis in this paper is based on trade interactions among the largest 100 firms (in terms of employees) primarily involved in the automobile sector in Japan (class 301 in the Japan Standard Industrial Classification, Rev. March 2002, at the three-digit level, which corresponds to 311 in the present classification). The largest manufacturers were selected based on how many employees they had in 2006. Such a selection of organizations that spans market boundaries has been described as an organizational community (Lomi and Pattison 2006). Tokyo Shoko Research interviewed firms’ representatives regarding their transaction partners. The informants were asked to name up to 24 of their main suppliers or buyers of goods or services. No other details about the interactions (such as the volumes of the transactions) were requested. The focus of this analysis is not the link content but whether major Japanese automotive firms distribute their business among many peers or only a few. Links to companies outside the top 100 firms within the industry are not considered in the present analysis. We use revenue per employee (RPE) and return on sales (ROS) as two measures of firm performance. For both measures of performance, we are interested in how supply network structures and the type of suppliers (e.g., labor-intensive original producers versus intermediates) relate to longer-term *changes* in clients’ performance.

First, we discuss RPE as a measure of performance. Naturally, larger firms are more likely to have larger revenues. Therefore, to analyze trends in the performance of firms of unequal sizes, RPE is used in this paper. Although RPE does not exactly measure the efficiency of a firm’s operations, increasing revenue trends for the same company can be generally interpreted as a performance improvement and decreasing revenues as a performance deterioration. However, caution is necessary when comparing the performances of firms in terms of RPE. Even within the same industry, RPE also reflects the type of business in which the firm is involved, including whether the firm is production or trade oriented. For example, upstream firms that manufacture their products internally are likely to have lower RPE because such production is labor-intensive. By contrast, trading firms and official first-tier suppliers that resell products manufactured by other firms are likely to have higher RPE.

For a given company in a certain line of business, an expansion of sales per employee indicates a shift to higher production with less labor. However, without controlling for all inputs, measuring only the change in outputs in relation to network dynamics does not sufficiently inform us whether interorganizational network structures contribute to operational efficiency. To estimate whether firms can increase their profits for a given volume of production by restructuring the networks through which they procure the parts required for this volume of production, we employ ROS.

Because the data do not include any information concerning the quantity of the transactions, we cannot control for the overall amount of components purchased through the supply chain to distinguish an increase in revenues from increased production volumes from an increase in the value of each product. Therefore, we include ROS as a measure of performance that adjusts for volumes.

RPE is characteristically skewed, and its natural log transformation is thus used in the present analysis, which is common in firm-level econometric studies. Because it is possible to model only the probability of discrete changes in the present modeling framework, log (revenues/employees) was rounded to the nearest integer value, which yielded an RPE scale with three performance categories: low, middle, and high (Table 3). For comparability, we also divided ROS in three categories according to the number of cases in the low (negative profits), high (ROS above 3%), and middle categories (all other firms), similar to the RPE scale. As a robustness check, we varied the number of categories and the cut-off points, which did not substantially change the results presented here. The sample is described in Table 1.

**Table 1:** The revenues and number of employees of the 100 largest firms in the Japanese automobile manufacturing sector

	Min.	Median	Mean	Max.	NA
Revenues in 2006 [thousands of yen]	7.39*10^6^	7.27*10^7^	3.88*10^8^	9.22*10^9^	1
Revenues in 2011 [thousands of yen]	5.00*10^6^	8.10*10^7^	4.02*10^8^	8.24*10^9^	8
Employees in 2006	800	1395	4316	65994	0
Employees in 2011	630	1580	4862	69310	7
RPE in 2006 [thousands of yen/person]	7393	52390	61220	143900	1
RPE in 2011 [thousands of yen/person]	6098	48960	57980	20560	8
logRPE 2006	8.908	10.87	10.88	11.88	1
logRPE 2011	8.716	10.8	10.81	12.23	8
Profit in 2006 [thousands of yen]	−5.26*10^8^	1.50*10^6^	6.12*10^6^	5.29*10^8^	2
Profit in 2011 [thousands of yen]	−3.97*10^7^	1.54*10^6^	4.59*10^6^	8.67*10^7^	10
ROS in 2006	−0.452	0.019	0.013	0.088	2
ROS in 2011	−0.113	0.016	0.019	0.152	11
Number of suppliers in 2006	0	1	4.5	45	
Number of suppliers in 2011	0	2	5.2	41	

A simple correlation test shows that the number of suppliers and RPE are strongly correlated (R = 0.5, *p* < 0.01). However, it is not possible to discern from the observed correlations alone whether accessing more suppliers leads to higher revenues, whether more successful companies tend to subsequently expand their supply chain, or neither. The methods introduced in the following section allow us to distinguish these mechanisms.

## Stochastic actor-oriented network modeling

### General introduction

Sophisticated statistical network models such as Exponential Random Graph Models have been developed for the analysis of complex network structures (Rank et al. 2010; Sosa et al. 2015; Trapido 2013) and applied to cross-sectional supply network data (Lomi and Pattison 2006). These models quantify the statistical prevalence of network micro-patterns, or “motifs” in networks. However, static models applied to cross-sectional data cannot untangle the mechanism of organizational behavior behind the dynamics of co-evolving interorganizational networks and organizational performance.

This study illustrates an application of dynamic models in which networks are gradually longitudinally constructed bottom-up by actors (representing organizations) making decisions concerning their organizational partners. This conceptualization corresponds to real-world interorganizational networks (Ahuja et al. 2009). We introduce stochastic actor-oriented models, which are statistical, parametric models of network evolution and diffusion (Snijders et al. 2010). Stochastic actor-oriented models have been successfully applied to interorganizational networks (van de Bunt and Groenewegen 2007). However, these models have only recently been extended to cope with the complexity of bidirectional interactions between networks and performance. Furthermore, stochastic actor-oriented models have become capable of robust goodness-of-fit tests, which can guide the model development and ensure its validity, which was not previously possible. These two novel aspects, i.e., (1) the co-evolution of interorganizational networks and organizational performance and (2) model validity, are emphasized in this article.

Variation of stochastic actor-oriented models has in recent years been applied to several trade network studies (Manger and Pickup 2016; Manger et al. 2012, Matous and Todo 2016, Prell and Feng 2016). For readers unfamiliar with the fundamentals of this method, we provide a short general introduction in the Appendix.

### Drivers of network and performance change

In stochastic actor-oriented models, network evolution is treated as a continuous-time Markov chain of single trading-link changes between observations. Between observations, each organization may receive one or more opportunities in a random order to change its suppliers by rearranging its outgoing ties, and the organization may also move up or down on the RPE or ROS scale. The model includes “rate effects” that regulate how frequently actors receive an opportunity to modify their outgoing ties and the frequency of changes in RPE or ROS. These rate effects depend on the number of observed changes in the data. Only one actor acts at a time, and coordination is not allowed. It is possible to allow actors with more links to make more changes in their networks, but this option did not improve the fit of the models in the present case. The parsimonious models presented here converged perfectly even without including such degree-dependent rate effects.

Each firm chooses its suppliers according to an objective function in which the desirability of each network configuration *x* is expressed from the viewpoint of actor *i* – as in generalized linear models – as a combination of hypothetically relevant network features *f*_*i*_(*β*, *x*) = ∑_*k*_ *β*_*k*_ *s*_*ki*_(*x*). A random component with a standard Gumbel distribution is added to the evaluation function. This component is included to respect the stochastic character of network evolution, which is a result of influences that are unrepresented by nodal or dyadic variables and of measurement errors. Thus, the actor does not necessarily choose the state with the highest utility, but such a choice is the most likely. When a firm has an opportunity to change its suppliers, the options are to create one new tie, delete one existing tie, or do nothing. An analogous but separate function is used to express the likelihood of an increase or decrease in RPE or ROS. This process enables us to untangle what comes first: network change or performance change?

Each effect *s*_*ki*_ in the model corresponds to possible reasons that a change in the organization’s network or performance might occur. These network evolution effects describe the organization’s network behavior, which considers the influence of the existing network structure and other organizations’ performance. The performance evolution effects describe the possible effects of the supply network’s structure on revenues. The preference for certain network patterns need not be conscious—actors may not be aware that they create network triangles when selecting suppliers that were recommended to them by their partners—the estimated effects merely quantify a statistical regularity. The explanations and mathematical formulas of effects *s*_*ki*_ are presented in Table 2.

**Table 2:** Formulas for s_ki_(x) selection effects in network x for ego i and alter j, other actors h, and actors’ attributes v. In the actor-oriented modeling framework, network links are directed from clients, who make the procurement decisions, to the suppliers that they select. Dashed arrows signify trading relationships that are likely to be created and maintained if the effect is positive

Effect name (Additional description)		Mathematical formula	Graphical representation
*1. Network dynamics*			
*1.1. Endogenous trade network interdependencies* Network → network			
Reciprocity (Favor firms that buy something from our firm)		${\displaystyle \sum_j{x}_{ij}{x}_{j i}}$	
Preference for firms with partners in common (i.e., firms within the same trading group)	Transitive triplets (Hierarchical cliques)	${\displaystyle \sum_{j, h}{x}_{ij}{x}_{j h}{x}_{hi}}$	
	Three-cycles (Non-hierarchical cliques)	${\displaystyle \sum_{j, h}{x}_{j i}{x}_{ih}{x}_{j h}}$	
	Common suppliers (Connect with firms that use the same suppliers)	$$ {\displaystyle \sum_j{x}_{ij}}{\displaystyle \sum_{\begin{array}{c}\hfill h\hfill \\ {}\hfill h\ne i, j\hfill \end{array}}\left({b}_0 x-\left|{x}_{ih}-{x}_{j h}\right|\right)} $$	
Number of second-tier suppliers (Connect with multiple primary suppliers through intermediaries)		# [j|*x*_*ij*_ = 0, *max*(*x*_*ih*_ *x*_*hj*_) > 0]	
Indegree popularity (Seek the most popular suppliers)		${\displaystyle \sum_j{x}_{ij}}{\displaystyle {\sum}_h{x}_{h j}}$	
Outdegree (Control for network density)		${\displaystyle \sum_j{x}_{ij}}$	
*1.2. Effects of firms’ performance z on supply network structures* Performance → network			
Client’s performance (High-performing firms connect with more suppliers)		${\displaystyle \sum_j{x}_{i j}{z}_i}$	
Supplier’s performance (Selecting high-performing firms as suppliers)		${\displaystyle \sum_j{x}_{ij}{z}_j}$	
Similarity of performance (Preference for firms with similar performance)		${\displaystyle \sum_j{x}_{ij}\left( si{m}_{ij}^z-\overline{si{m}^z}\right)}$	
*2. Performance dynamics*			
Linear performance trend (Baseline revenue trend)		*z*_*i*_	
Performance → revenues			
Quadratic performance trend (The effect of current performance on the future performance trend)		${z}_i^2$	
Network → performance			
(A) Topology: The effect of the number of suppliers on the future performance trend		${z}_i{\displaystyle \sum_j{x}_{i j}}$	
B Flow: The effect of the suppliers’ performance on the performance trend		${\displaystyle \sum_j{x}_{ij}\left( si{m}_{ij}^z - \overline{si{m}^z}\ \right)}$	

*Note*: *x*_*ij*_ = 1 if there is a directed tie from *i* to *j* and 0 otherwise ^b^ $\overline{si{ m}^z}$ is the mean of all similarity scores, which are defined as $s i{m}_{i j}^z=\frac{\varDelta -\left|{z}_i-{z}_j\right|}{\varDelta}$ with *Δ* = *max*|*z*_*i*_ − *z*_*j*_|

The goal of the simulation is to estimate the relative weights *β*_*k*_ for the statistic *s*_*ki*_. Parameter estimates can be used to compare how attractive various network configurations are and the likelihood of any change in performance while controlling for other exogenous and endogenous effects. The sign of *β*_*k*_ indicates the likely direction of network or revenue change, and the relative magnitudes can be interpreted similarly to parameters of multinomial logistic regression models in terms of the log-probabilities of changes among which the actors can choose. Specifically, the estimates are the log odds ratios of procuring parts from a supplier described by the effect or a one-step improvement in performance on the RPE or ROS scale. Although it is possible to allow different values of *β*_*k*_ for creating new links and maintaining old links (e.g., to capture the inertia of maintaining old interorganizational relationships), this distinction did not improve the fit of the presented models, suggesting that the relationships were relatively fluid in the observed period.

In summary, the model uncovers the network “microdynamics” (Ahuja et al. 2011), i.e., the actors’ tendencies to create and maintain certain network micro-patterns. This process is distinct from statistically assessing only the prevalence of such patterns in the data. For example, the presence of cohesive groups is typically expressed in network terms as a high presence of triangles (i.e., partners of partners are also partners). However, the changing number of triangles alone might not be always driven by the actors’ preference for partners from the same trading clique. Different micro-mechanisms can produce similar patterns in the data. Trivially, the number of incidental triangles will increase with more trading links in the network. Alternatively, there may be substantially important reasons for the changing numbers of basic network motifs. For example, if most firms across the network seek the best supplier regardless of keiretsu boundaries, the best supplier will emerge as a new hub to which many nodes are connected. Thus, many firms in the network will be connected in two steps through the hub. Then, any new links between clients of this hub will create triangles, even if these new links were not motivated by the presence of this mutual business partner. By iteratively simulating the network evolution with actors’ varying supply chain management strategies (such as the preference for popular suppliers), we aim to determine the real drivers of the network and performance dynamics that most faithfully replicate observable reality.

### Simulation and model validation

The estimation of stochastic actor-oriented models can be executed in the RSiena package in R software (Ripley et al. 2016). The method of moments, which depends on thousands of iterative computer simulations of the change process, is used to estimate the parameters *β*_*k*_ that enable the reproduction of interorganizational network evolution within the observed period. There is one target statistic for each estimated effect (for example, the number of ties in the network corresponds to the outdegree effect, the number of reciprocated ties corresponds to the reciprocity effect, and the amount of change in the network corresponds to the rate function). The presented models all converge with *T*-ratios, quantifying the deviations between the simulated and observed values of the target statistics, between −0.1 and 0.1, with the overall maximum convergence ratios below 0.2, indicating excellent model convergence (Ripley et al. 2016). In the final stage of the simulation, the standard errors of the estimated parameters are computed by the finite difference method, based on the sensitivity of the target statistics to *β*_*k*_.

Statistical models must be evaluated after parameter estimation, and goodness of fit must be assessed. Without goodness of fit assessment, the reliability of all models, including stochastic actor-oriented models, is doubtful. We assess the overall validity of the model by the goodness of fit of the simulated networks’ macro-characteristics. We compare the goodness of fit of the simulated networks and the actual networks at time 2 in terms of indegree distribution (i.e., the distribution of the number of clients), outdegree distribution (i.e., the distribution of the number of suppliers), geodesic distance distribution (quantifying the overall connectivity of the network), and triadic census (quantifying the distribution of all combinations of directed triangles in the network).

**Fig. 1:**
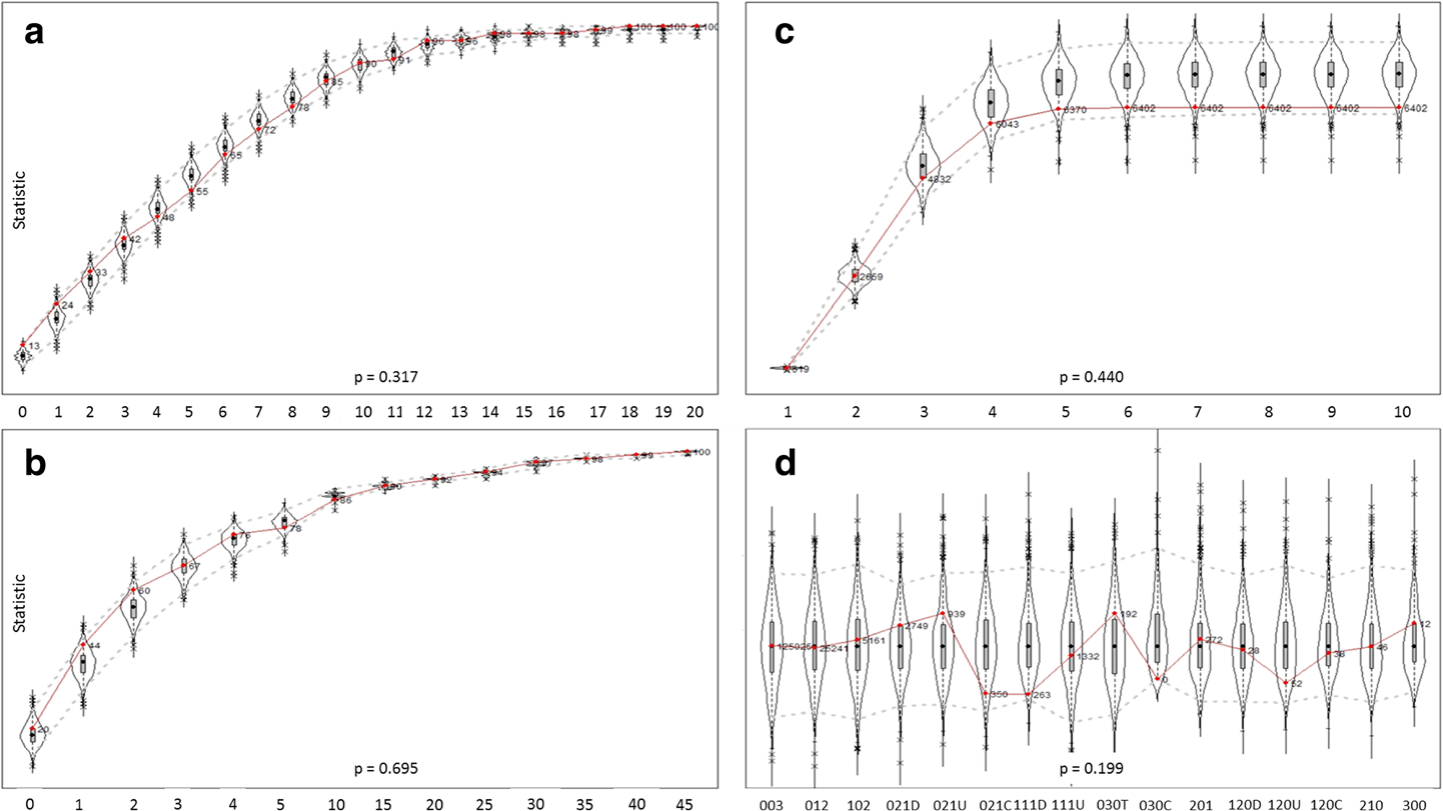
**a** Distribution of the number of clients per firm; **b** distribution of the number of suppliers per firm; **c** geodesic distance distribution; **d** triadic census

The violin plots (Hintze and Nelson 1998) in Fig. 1 represent the kernel density distribution of the statistic, and the red lines depict the cumulative distribution of the observed values. The dotted gray lines designate a point-wise 90% relative frequency band for the simulated data. Qualitatively, the fit is considered acceptable if the observed values (red lines) fall within this region. The overall fit of each diagram is also quantitatively summarized in terms of *p*-values below the diagrams, which were obtained by combining the vector of statistics using Mahalanobis distance. Values above 0.05 indicate acceptable fit. Standard labeling is used for the classes of the triad census (Wasserman and Faust 1994), which is scaled and centered to maximize the visibility. Only the goodness of fit of the ROS Flow model is presented in the figure for illustration. The diagrams for the other three models presented in this paper display similarly good fit.

### Resulting model

The effects that needed to be included in the model to correctly reproduce the observed network dynamics are explained in Table 2. The reciprocity effect in Section 1.1. of Table 2 represents a fundamental tendency of many real-life networks to reciprocate links among actors (Wasserman 1980). Reciprocity is common even in supply chains, which were traditionally understood as linear chain-like entities, in which resources flow in one direction (Matous and Todo 2016). Organizations are typically more likely to obtain resources mutually from one another than from a random, unrelated organization in the dataset.

Transitive triplets, three-cycles, and common supplier effects represent different types of transitive closure or clique formation in directed networks. The number of partners that two interlinked organizations have in common reflects the structural embeddedness of their relationship (van de Bunt and Groenewegen 2007). A potential supplier may be preferred if other business partners also use the supplier. The formulas for the transitive closure effects in Table 2 quantify how many triangles are closed by a link from *i* to *j.* The number of the respective types of triangles multiplied by the obtained weights *β*_*k*_ equals the utility such a link adds to the actor and translates into the probability of creating and maintaining this link. The effect entitled “Number of second-tier suppliers” tests whether organizations prefer suppliers that mediate access to a large number of second-tier suppliers. Furthermore, organizations may imitate other organizations in choosing their suppliers and prefer more central organizations (Ahuja et al. 2009, Hoetker et al. 2007, Stuart 1998). Such tendencies are captured by the indegree popularity effect. Indegree popularity quantifies “network-related status” (van de Bunt and Groenewegen 2007) and controls for the preference to connect to other popular organizations. In general network terms, this tendency is also called preferential attachment and addresses the tendency of hubs to emerge in interorganizational data. Firms do not have resources to create an unlimited number of interorganizational links (Ahuja et al. 2009). The outdegree effect controls for the number of links in the network. The effects in Section 1.2 of Table 2 jointly test whether organizational performance influences network evolution. Finally, the effects in Section 2 control for the overall change in performance (“Baseline performance trend”) and for the performance distribution among firms (“Quadratic performance trend”). The last two effects assess the consequences of networks for organizational performance in terms of (A) topology and (B) flow. By contrast, the topology effect tests whether organizational performance changes if an organization’s number of suppliers changes (regardless of the suppliers’ performance); the flow effect tests whether partnering organizations tend to converge in their performance.

## Results

### Descriptive statistics

The changes in the supply networks and performance between 2006 and 2011 are summarized in Table 3. Whereas 388 supply links remained unchanged, 169 changes occurred, including 131 newly created supply links and 38 severed links. The overall supply network density among these 100 largest automobile firms increased from 0.045 to 0.059, as the average number of suppliers of each firm within the sample increased from 4.5 to 5.9. Six of the top 100 firms from 2006 are not present in the 2011 data. This proportion of missing values is within the limits tolerated in this modeling framework and should not impair the reliability of the results (Ripley et al. 2016). The values for the links among the six firms were imputed with values of the same dyads in the previous observation, i.e., links with missing data were considered unchanged. Table 2 displays values based on the raw data, whereas the metrics in Table 3 are calculated following imputation. In terms of the RPE categories, 12 firms improved their ranks and 20 firms decreased their ranks. In terms of the ROS categories, 24 firms improved their ranks and 39 firms decreased their ranks.

**Table 3:** Descriptive results: changes of suppliers between 2006 and 2011, distribution of firms by revenue categories and by revenue dynamics

	Count
Network dynamics	
Whole network density in 2006	0.045
Whole network density in 2011^a^	0.059
Average number of suppliers in 2006	4.50
Average number of suppliers in 2011^a^	5.88
Preserved supply relationship	388
New suppliers	131
Abandoned suppliers	38
Total of changes	169
Jaccard index	0.697
Missing links in 2006	0%
Missing links in 2011	11.7%
RPE performance categories	
Low revenue firms in 2006 (logRPE < 10.5)	21
Middle revenue firms in 2006 (10.5 < =logRPE < 11.5)	62
High revenue firms in 2006 (logRPE > =11.5)	16
NA in 2006	1
Low revenue firms in 2011 (logRPE < 10.5)	25
Middle revenue firms in 2011 (10.5 < =logRPE < 11.5)	55
High revenue firms in 2011 (logRPE > =11.5)	12
NA in 2011	8
ROS performance categories	
Loss-making firms in 2006 (ROS < 0)	12
Middle return firms in 2006 (0 < =ROS < =0.03)	59
High return firms in 2006 (ROS > 0.03)	29
Loss-making firms in 2011 (ROS < 0)	27
Middle return firms in 2011 (0 < =ROS < =0.03)	48
High return firms in 2011 (ROS > 0.03)	25

^a^The network metrics in this table were calculated after the imputation of 2006 values for the 11.7% missing values in 2011

### Modeling results

The effects describing the network dynamics are in the upper part of Table 4, and the lower part of the table describes the performance dynamics. All these effects are jointly considered in the model. The results for the network dynamics part of the model are qualitatively similar for all the presented specifications. In the following discussion, we illustrate the meaning of the estimates of network dynamics by citing the values of RPE topological model unless otherwise noted.

**Table 4:** Stochastic actor-oriented model: the network dynamics component of the model estimates the log odds of procuring parts between a client and supplier embedded in network structures and characterized by performance described by the estimated effects; the revenue dynamics component of the model estimates the log odds of increasing productivity by one step on the RPE or ROS scale

	RPE				ROS			
	Topology		Flow		Topology		Flow	
Effect name	Parameter estimate	Std. error	Parameter estimate	Std. error	Parameter estimate	Std. error	Parameter estimate	Std. error
*1. Network dynamics*								
*1.1. Endogenous trade network interdependencies* Network → network								
Reciprocity	2.120*	0.308	2.151*	0.295	2.741*	0.252	2.204*	0.454
Transitive triplets	0.116	0.071	0.078	0.056	0.089	0.060	0.087	0.053
Three-cycles	0.139	0.135	0.150	0.143	0.153	0.144	0.153	0.154
Same suppliers	−0.063*	0.025	−0.064*	0.023	−0.073*	0.027	−0.067*	0.020
Number of second-tier suppliers	−0.137*	0.038	−0.138*	0.044	−0.132*	0.033	−0.121*	0.023
Indegree popularity	0.113*	0.027	0.116*	0.024	0.118*	0.024	0.117*	0.028
Outdegree	−2.593*	0.356	−2.699*	0.314	−2.635*	0.337	−2.754*	0.212
*1.2. Effects of firms’ performance z on supply network structures* Performance → network								
Client’s performance	1.135	1.303	−0.327	1.831	−0.982	1.361	−0.688	1.336
Supplier’s performance	0.049	0.461	0.029	0.774	0.206	0.445	0.195	0.624
Similarity in performance	−3.022	1.776	−3.953	3.562	−1.451	1.587	−1.231	2.410
*2. Performance dynamics*								
Baseline performance trend	−0.673*	0.231	−0.174	0.180	−0.058	0.167	−0.051	0.194
Performance → performance								
Quadratic revenue trend	−1.267*	0.373	−1.296*	0.470	−0.248	0.278	0.006	0.382
Network → performance								
Topology: The effect of the number of suppliers on the future performance trend	0.101*	0.033			−0.002	0.012		
Flow: The effect of the suppliers’ performance on the client’s performance trend			−0.217*	0.110			0.209	0.212

*p < 0.1

First, we report the effects describing the endogenous network evolution in Part 1.1 of Table 4. As expected, the reciprocity effect is positively significant, which indicates that bidirectional trade between pairs of firms is more common than expected by chance, after controlling for other effects. In other words, a firm is more likely to procure goods or services from a firm that procures goods or services from it, *ceteris paribus*. The estimates of the model can be interpreted in the same manner as the results of a logistic regression. As previously explained, the objective function quantifies the desirability of different interorganizational networking strategies (specifically, the log odds of alternative supply chain configurations), and procuring goods from an existing client increases the value of the objective function by 2.120 (Table 4, 1.1), indicating that a firm that is given the opportunity will prefer to procure goods from a firm that also procures goods from it with 8 to 1 odds, compared with otherwise equivalent alternatives (because e^2.12^ is approximately 8).

Next, three effects describe the tendency of actors to form triangular elements in the supply networks by preferring to form and maintain links with partners from the same cliques with many partners in common. The first two effects are transitive triplets and three-cycles. The transitive triplets are triangular network motifs in which supplies flow from one side to the other; the three-cycles represent cliques with cyclical flows. Both effects are insignificant. Furthermore, the following negatively significant “same suppliers” effect also operates against network closure. (This effect corresponds to the “balance” effect in the Siena modeling framework.) The negative estimate of this effect indicates that firms were likely to abandon links to partners that procured supplies from the same partners and that they sought firms that were not yet accessed by their existing partners.

If firms preferred to retain suppliers from the same densely interconnected business groups with many partners in common, we would expect the effects representing transitive closure (transitive triplets, three-cycles, same suppliers) to be positively significant. Although network closure effects are typically important drivers of network evolution (Ripley et al. 2016), none of the triadic effects were significant in the present data. The lack of significance of the transitive closure and three-cycles and the negative significance of the “same suppliers” indicate that it was possible during this period to disconnect from organizations even if they had many partners in common and to connect to otherwise unrelated suppliers in different cliques. The goodness-of-fit tests show that the lack of cliquish tendencies is modeled correctly in the present simulations (Fig. 1). The only significant effect related to transitive closure is the “number of second-tier suppliers,” which is discussed next.

The effect “number of second-tier suppliers” is negatively significant and indicates that organizations preferred to abandon intermediates that provided indirect access to multiple second-tier suppliers. This result means that the more suppliers that a firm has, the less attractive it becomes to other firms as a supplier. For example, severing a contract with an intermediary that provides indirect access to 10 s-tier suppliers contributes 10*0.137 to the firm’s objective function, which describes the log odds of different supply chain configurations. Thus, if an assembler had to choose between (1) procuring supplies from an intermediate that buys parts from 10 producers or (2) procuring directly from an original producer that has no other suppliers in the network, the odds ratios of choosing option 2 versus option 1 would be exp(0*(−0.137)-10*(−0.137)), i.e., 3.9:1. In other words, if the two options were hypothetically equivalent in terms of all the other effects, the assembler would choose the original producer instead of the intermediate, with an approximately 80% probability.

Both dismissing first-tier suppliers that broker access to many second-tier suppliers and connecting directly with second-tier suppliers (thus converting them into first-tier suppliers) decrease the number of second-tier suppliers and are thus supported by this negatively significant effect. (These two ways in which the “number of second-tier suppliers” effect operates are depicted by the two diagrams for this effect in Table 2.) Note that, although the main transitive closure effects were not positively significant, creating links to second-tier suppliers may be an alternative micro-mechanism producing transitive closure, if the original link to the first-tier supplier is also preserved. However, creating a secondary path to a supplier previously accessed through a broker is less likely than severing the contract with the broker for the following reasons. A new direct link to a second-tier supplier contributes only 0.137 to the firm’s utility because this link transforms the second-tier supplier into a first-tier supplier and, in turn, decreases the number of second-tier suppliers by one. By contrast, severing the link with the broker contributes 0.137 multiplied by the number of the broker’s suppliers to the objective function. Additionally, duplicate paths to the same suppliers via brokers are also discouraged by the negative “same supplier” effect.

The firms in the sample sought the most popular suppliers with many clients, which is captured by the indegree popularity effect.

From the joint results regarding the dynamics of interfirm network structures above, we can state that the first structural embeddedness hypothesis (H1) is not supported.

The outdegree effect estimates and controls for the number of suppliers that firms in the sample connected to within this period. The negative estimate is expected because most firms procure supplies from only a small fraction of firms within the sample. A procurement link becomes statistically likely only if the total log odds of a link become greater than zero by the activation of other positive network effects, such as by connecting to a popular supplier.

The model also accounts for the potential influence of firms’ changing performance on their subsequent supply chain management strategy and on their attractiveness as suppliers, i.e., for the possibility that similarity (or dissimilarity) in performance between two firms may influence the probability of trade between them. These three effects all test the influence of performance on networks, and all are insignificant in the final model, as shown in Part 1.2 of Table 4. Notably, in an incomplete model that did not account for the possible opposite direction of causality (the effect of networks on revenues), clients’ performances appeared to have a positively significant effect on diversifying the supplier base. Thus, when the possibility of causality in both directions is not considered, it appears that good performance is followed by an increase in the number of suppliers. However, the presented specifications show that the opposite causal mechanism more faithfully reconstructs the observed dynamics. Specifically, improved performance appears to be a consequence of expanded supply chains to a broad range of first-tier firms rather than the reverse.

Now, we turn to organizational performance, specifically the revenue dynamics (Part 2 of Table 4). The negatively significant baseline linear revenue trend combined with the negatively significant quadratic effect signifies that previously high RPE firms were more likely to exhibit worse subsequent performance. This relationship does not appear in the ROS models.

Having controlled for potential effects of past performance on current performance trends, the last part of the model examines the effect of supply networks on firms’ performance based on two alternative specifications. The topological specification examines whether the number of suppliers influences the performance trend. The result is positively significant for RPE (0.101) but close to zero for ROS (−0.002). The ROS estimate is not significantly different from zero despite the small standard error associated with this estimate. Approximate 95% confidence intervals calculated as the estimate ± 1.96* standard error for the additional effect of each supplier on the log odds of performance increase in terms of RPE and ROS are 0.036 to 0.166 and −0.025 to 0.022, respectively. We can say with reasonably high precision that while a wider range of suppliers increases RPE, it does not increase ROS. This result contradicts the second structural embeddedness hypothesis (H2).

The flow specification tests whether the performance of the suppliers has an impact on clients’ performance. This specification produces negatively significant results for RPE, suggesting that obtaining supplies from high-RPE firms has a negative effect on the assembler’s RPE over the long term. The estimate for ROS is of a similar magnitude in absolute value but positive. However, the standard error associated with the ROS estimate is higher, and we cannot confidently state whether suppliers’ ROS has an effect on clients’ ROS. This result contradicts the third structural embeddedness hypothesis (H3).

Overall, the combined results for performance in terms of RPE and ROS suggest that firms that diversified and increased the number of their direct partners were able to produce and sell more units at their original margins rather than eliminating waste and adding more value per a unit of production by accessing new suppliers.

## Discussion and practical implications

It is directly observable that the largest Japanese manufacturers increased the number of direct supply connections with one another during this period and, as a result, increased supply network density across the industry. However, stochastic actor-oriented modeling did not reveal any particular preference for creating and maintaining dense connections within separate network cliques, which would be expected if keiretsu considerations had continued to constrain procurement strategies. The negatively significant and insignificant estimates of those effects representing transitive closure in triads contrast with findings from other network studies and, in particular, with the positively significant tendencies toward closure uncovered by the same methods on the same type of data for the largest Japanese firms from all industries (Matous and Todo 2014). In the wake of recent scandals regarding collusion among suppliers and price fixing in the automobile industry (Shirouzu and Shiraki 2014), it is plausible that Japanese assemblers and pre-assemblers are accessing diverse groups of suppliers to enable the shifting of business if necessary. These supply chain management changes might have become technically easier to implement as modularization – which allows for direct substitution of parts from different suppliers –has become prevalent in the automobile industry (Corswant and Fredriksson 2002). Strong long-term relationships are less useful when dealing with substitutable modular components (Hoetker et al. 2007, Novak and Eppinger 2001).

Despite the fact that disintermediation decreases the number of vertical steps in the supply chain, and supplier diversification decreases exposure to common risks (Babich et al. 2007; Wan and Beil 2009), quality control and safety assurance may become more challenging in the new, more fluid environment of production networks. This new challenge may prove particularly demanding for Japanese automakers with extremely slim procurement departments. Our finding that car manufacturers seek hub suppliers across keiretsu boundaries resonates with the concerns voiced by a white paper from the Japanese Ministry of Trade, Economy, and Industry (METI 2011). This white paper, which was published soon after the Great East Japan Earthquake in 2011, reported that manufacturing procurement in Japan has acquired a “diamond structure,” characterized by the concentration of supply links on certain key original producers of parts and materials. The implication of the new macro-structure, which resulted from the micro-processes analyzed in this paper, is that negative temporary shocks can now propagate throughout entire supply networks regardless of keiretsu boundaries when such suppliers suffer damage.

The findings of the negative influence of suppliers’ RPE on clients’ RPE and the positive influence of the number of suppliers on clients’ RPE merit attention. Suppliers may have high RPE if they do not engage in labor-intensive production of the parts they are selling. According to the former vice-executive director of Toyota, this description fits the typical keiretsu first-tier suppliers (Shirouzu 2015). Toyota and the other major Japanese assemblers that followed the Toyota method have traditionally engaged in business only with a small number of permanent first-tier suppliers and have expanded production only through first-tier suppliers by allowing them to procure higher volumes of materials from other manufacturers and to resell them to Toyota (Wada 1992). Although such keiretsu arrangements have long been considered the source of the high competitiveness of Japanese manufacturers (Aoki 1990; Dyer 1996), the results of this paper overall suggest the following: (1) Japanese automakers may be moving away from this strategy by bypassing intermediaries, reaching across network cliques, and expanding the number of their direct suppliers; (2) this strategy enables them better to ramp-up production compared to the originally preferred exclusive procurement through a few designated intermediates. Specifically, the present method enables us to observe that diversification of supply networks led to increased revenues per employee, as opposed to increased demand followed by an expansion of the supply base.

From the topological perspective, these findings relate to opinions in the organizational literature on “the dark side of social capital,” which note that networks may impede the performance of actors who are “imprisoned” in old, ineffective relations (Gargiulo and Benassi 2000). The results are also in line with Burt’s position on social capital as an outcome of reaching to diverse cliques across “structural holes” (Burt 1995) rather than bonding within one’s own clique (Coleman 1990).

From the network flow perspective, the literature on diffusion in organizational studies has demonstrated how interorganizational networks may lead to the homogenization of practices (Davis and Greve 1997). Furthermore, based on the view that, within Japanese business groups, high-performing organizations pull forward low-performing partners by risk sharing and revenue redistribution (Khanna and Yafeh 2005), we would assume that partnering with high performers has positive implications for productivity.

The present method uncovered an opposite case of interorganizational network influence: the studied corporations tended to diverge from their partners in terms of revenues per employee. This may be a case of networks being a source of heterogeneity of practices (organizations can outsource less-productive, labor-intensive activities if they find suitable partners for these tasks), and such “negative diffusion” effects can be uncovered with the introduced method, while controlling for the effects of organizational agency.

Importantly, the ROS analysis showed that the increase in revenues through the described changes in supply change management is not accompanied by increasing margins. The most likely interpretation of the results is that reaching a wider range of suppliers enabled the successful automakers to increase the number of units sold per employee rather than increasing the value added per unit. Although such reforms may catalyze business expansion, restructuring interorganizational links does not suffice to increase operational efficiency. Intraorganizational process improvement cannot be neglected if the goal is to increase the value added for each unit of production. Rigorous methods are needed to distinguish when interorganizational network structure matters and when it does not.

## Closure: too much of a good thing?

Industrial production structures are built bottom-up from interactions among individual organizations that require one another’s material and non-material resources. Individual interorganizational relationships, from which the structure of the entire industrial production system emerges, have been explained in terms of social context (Granovetter 1985), knowledge and organizational learning (Ahmadjian and Lincoln 2001; Podolny et al. 1996), status (Podolny 1993), trust (Bradach and Eccles 1989; Gulati 1995), transaction costs (Jones 1997), and market and hierarchies (Simon 1991; Williamson 1991).

Contrary to the structural embeddedness hypotheses, within the presently analyzed sample and time frame, more embedded firms were less successful, and the whole system moved toward less embeddedness. There is a limit to which strong ties benefit organizational performance (Todo et al. 2016). When a certain limit of embeddedness is crossed, the flow of new knowledge into the network decreases, feelings of obligation override economic considerations, and change becomes difficult (Glasmeier 1991; Uzzi 1996). In such a situation, switching to ties with less mutual obligations enables producers to economically maintain more links scattered across the market among actors who may provide access to new resources and information (Uzzi and Lancaster 2003). The present analysis suggests that Japanese automakers have found themselves on the side of too much embeddedness, corrected for it, and benefited from that.

Although the term “organizational network” was traditionally reserved for embedded cliquish systems of interorganizational relations, systems of fluid and diffused market relations can also be analyzed as networks. It is possible to understand how configurations of industrial networks evolve and how these configurations influence the ways in which the activities of each organization aggregate at the collective level due to novel dynamic approaches that connect the micro and macro levels (Ahuja et al. 2011).

The argument for shifting the analytical focus of interorganizational research from individual interactions to the broader micro-mechanism from which the complex macro patterns of interorganizational resource exchange emerge is not new (Bradach and Eccles 1989). However, the transition has not been fully possible with traditional analytical methods because of the endogenous character of mutually interdependent changes of interorganizational relationships. Organizations form one another’s dynamically changing environment and may influence one another’s performance in unexpected ways. Every new relationship modifies the existing network, prompting an endogenous dynamic between organizational action and network structure (Gulati and Gargiulo 1999). Empirical research could not until recently realistically address this complexity and instead treated networks only as either dependent or independent variables because of the lack of suitable longitudinal data and dynamic modeling methods (Ahuja et al. 2011).

Focusing on “network microdynamics” (Ahuja et al. 2011), i.e., dynamic micro-structures of choices that individual organizations systematically make regarding their partnerships with other organizations, we illustrated one way to develop models that realistically explain interorganizational network and organizational performance coevolution. The introduced models integrate the analysis of organizational agency and structure and enable both structuralist and connectionist approaches to the analysis of network consequences. We believe that novel methods, such as those presented here, will enable researchers to better understand real-world interactions among organizations and their consequences.

## Appendix

### Introduction to stochastic actor-oriented models

Stochastic actor-oriented models are a special type of Agent-Based Model (ABM). One of the distinguishing features of these models is that standard ABMs cannot typically be used for analyzing empirical data but instead are used to explore stylized, hypothetical what-if scenarios (Fioretti 2012). Moreover, whereas ABMs also produce network structures of agents’ interactions, such structures are normally impossible to validate with real-world data, which impedes the acceptance of ABMs in organizational research.

Stochastic network models are used to analyze binary network data, which are becoming increasingly available and can address substantively important issues, such as the presence or absence of interorganizational partnerships. Stochastic actor-oriented models begin with a real network and a real distribution of actor characteristics at time 1 and, by iterative simulations, work to find stochastic “rules” of actors’ behavior and performance changes that will lead the network to restructure step-by-step into the shape observed at time 2. The process of searching for the “rules” underlying actors’ behavior consists of specifying a variety of theoretically meaningful effects in the model, which are introduced in the next sub-section, and estimating the strength of the effects to lead the evolution of the network at time 1 to a network that fits the observed network at time 2 in terms of its micro-characteristics (such as the number of closed triangles). The strength of the effects corresponds to the probability that an actor will create and maintain ties in the direction specified by the effect. Finally, the simulated networks are compared to the observed reality at time 2 in terms of macro-characteristics that were not directly fit in the iterative simulations. Thus, the model is validated at both the micro-level of agents’ interactions and at the macro-level of the structures generated. This enables a level of realism unattainable by the vast majority of ABMs (Fioretti 2012).

Stochastic actor-oriented models were originally applied to directed expressive networks of individuals, such as friendships within organizations (Schulte et al. 2012). We argue that the assumptions of these models regarding the evolution of directed networks are suitable for networks of resource exchange among organizations. In the case of supply networks, a link from A to B means that A chooses B as a supplier (By contrast, in the case of friendship networks, a link from A to B is interpreted as A “nominating” B for friendship, the meaning of which is slightly less straightforward).

Another important aspect of stochastic actor-oriented modeling is that actors quantify the potential value of links to all other actors in the dataset at every step. While this assumption may make models of informal interpersonal networks within large groups of people less realistic (employees of large organizations may not frequently re-assess all other employees for potential friendship), it is much more plausible that procurement managers compare the pros and cons of all available suppliers.
